# Supplementary material for: Artificial Intelligence in Risk Stratification and Outcome Prediction for Transcatheter Aortic Valve Replacement: A Systematic Review and Meta-Analysis
Source: J Pers Med. 2025 Jul 11;15(7):302. doi: 10.3390/jpm15070302 (PMC12298983; doi:10.3390/jpm15070302)
Supplement: Supplementary file 1 [file jpm-15-00302-s001.zip › Supplementary Table S2.pdf]

Search	Query	Results (No.)
PubMed	("transcatheter aortic valve implantation"[Title/Abstract] OR "TAVI"[Title/Abstract] OR "TAVR"[Title/Abstract] OR "transcatheter aortic valve replacement"[Title/Abstract]) AND ("Artificial Intelligence"[Title/Abstract] OR "AI"[Title/Abstract] OR "Machine Learning"[Title/Abstract] OR "Deep Learning"[Title/Abstract] OR "Neural Network"[Title/Abstract] OR "Random Forest"[Title/Abstract] OR "Gradient Boosting"[Title/Abstract] OR "Support Vector Machine"[Title/Abstract] OR "Predictive Modeling"[Title/Abstract] OR "Supervised Learning"[Title/Abstract] OR "Unsupervised Learning"[Title/Abstract] OR "Reinforcement Learning"[Title/Abstract] OR "Algorithmic Modeling"[Title/Abstract] OR "Computational Intelligence"[Title/Abstract] OR "Bayesian Network"[Title/Abstract] OR "Big Data"[Title/Abstract] OR "Data Mining"[Title/Abstract] OR "Predictive Analytic"[Title/Abstract] OR "Decision Tree"[Title/Abstract] OR "Cox Model"[Title/Abstract] OR "Proportional Hazards"[Title/Abstract] OR "Survival Analysis"[Title/Abstract] OR "Regression"[Title/Abstract] OR "Elastic Net"[Title/Abstract] OR "Lasso"[Title/Abstract] OR "Ridge"[Title/Abstract] OR "Regularization"[Title/Abstract] OR "Generalized Linear Model"[Title/Abstract] OR "GLM"[Title/Abstract] OR "Multivariate Regression"[Title/Abstract])	1,766
Embase	('transcatheter aortic valve implantation':ti,ab OR 'tavi':ti,ab OR 'tavr':ti,ab OR 'transcatheter aortic valve replacement':ti,ab) AND ('ai':ti,ab OR 'artificial intelligence':ti,ab OR 'ml':ti,ab OR 'machine learning':ti,ab OR 'dl':ti,ab OR 'deep learning':ti,ab OR 'neural network':ti,ab OR 'random forest':ti,ab OR 'gradient boosting':ti,ab OR 'support vector machine':ti,ab OR 'predictive modeling':ti,ab OR 'supervised learning':ti,ab OR 'unsupervised learning':ti,ab OR 'reinforcement learning':ti,ab OR 'algorithmic modeling':ti,ab OR 'computational intelligence':ti,ab OR 'bayesian network':ti,ab OR 'big data':ti,ab OR 'data mining':ti,ab OR 'predictive analytics':ti,ab OR 'decision tree':ti,ab OR 'cox model':ti,ab OR 'proportional hazards':ti,ab OR 'survival analysis':ti,ab OR 'regression':ti,ab OR 'elastic net':ti,ab OR 'lasso':ti,ab OR 'ridge':ti,ab OR 'regularization':ti,ab OR 'generalized linear model':ti,ab OR 'glm':ti,ab OR 'multivariate regression':ti,ab)	5,521
Total		7287

Supplementary Table S2. Search terms of databases
