# Supplementary material for: Artificial Intelligence in Risk Stratification and Outcome Prediction for Transcatheter Aortic Valve Replacement: A Systematic Review and Meta-Analysis
Source: J Pers Med. 2025 Jul 11;15(7):302. doi: 10.3390/jpm15070302 (PMC12298983; doi:10.3390/jpm15070302)
Supplement: Supplementary file 1 [file jpm-15-00302-s001.zip › Supplementary Table S3.pdf]

Study	TRIPOD Items																									
	1	2	3a	3b	4a	4b	5a	5b	6a	8	9	10b	10d	10e	12	13a	13b	14a	15b	16	17	18	19a	19b	20	21	22
Zusman, 2017	+	+	+	+	-	+	+	+	+	-	-	-	+	NA	NA	+	-	+	-	+	NA	+	+	+	+	+	+
Vejpongsa, 2018	+	+	+	+	+	+	+	+	+	-	+	+	+	NA	+	+	-	+	+	+	NA	+	+	+	+	NA	+
Hoffmann, 2019	-	+	+	+	-	+	+	+	+	-	-	+	-	NA	NA	+	+	-	+	+	NA	+	+	+	+	+	+
Lopes, 2019	+	+	+	+	+	+	+	+	+	-	-	+	+	NA	NA	+	+	+	+	+	NA	+	+	+	+	-	+
Hernandez-Suarez, 2019	+	+	+	+	+	+	+	+	-	-	+	+	+	NA	NA	+	+	+	+	+	NA	+	+	+	+	-	+
Tsushima, 2020	+	+	+	+	+	+	+	+	+	-	-	+	+	NA	NA	+	-	+	+	+	NA	+	+	+	+	NA	+
Abdul Ghffar, 2020	+	+	+	+	+	+	+	-	+	-	-	+	+	NA	NA	+	+	+	+	+	+	+	-	+	-	-	-
Gomes, 2020	+	+	+	+	+	+	+	+	+	-	+	+	+	NA	NA	+	+	+	+	+	NA	+	+	+	+	+	+
Truong, 2021	+	-	+	+	+	+	+	+	+	-	+	+	+	NA	NA	-	-	+	+	+	NA	+	+	+	+	+	+
Agasthi, 2021	+	+	+	+	+	+	+	-	+	-	+	+	+	NA	NA	NA	+	+	+	+	NA	+	+	+	+	-	+
Penso, 2021	+	+	+	+	+	+	+	-	+	-	NA	+	+	NA	NA	+	+	+	+	+	+	+	+	+	+	-	-
Lopes, 2021	+	+	+	+	+	+	+	+	+	-	+	+	+	+	NA	+	+	+	+	-	+	+	+	+	+	+	+
Okuno, 2021	+	+	+	+	+	+	+	-	+	-	-	+	+	NA	NA	+	+	+	-	+	NA	+	+	+	-	+	-
Mamprin, 2021, A	+	+	+	+	+	+	-	-	-	-	+	+	-	NA	+	+	-	-	+	+	NA	+	+	-	-	-	+
Mamprin, 2021, B	+	+	+	+	+	+	+	-	-	-	+	+	+	NA	NA	+	-	-	+	+	+	+	+	-	+	-	+
Galli, 2021	+	+	+	+	+	+	+	+	+	-	+	+	+	NA	+	+	+	+	+	+	NA	+	-	+	+	-	+
Aquino, 2022	+	+	+	+	+	+	+	+	+	-	+	+	+	NA	NA	+	+	+	+	+	NA	+	+	+	+	-	+
Bansal, 2022	+	+	+	+	+	+	+	+	+	+	+	+	+	NA	NA	+	+	+	+	+	NA	+	+	+	+	-	+
Evertz, 2022	+	+	+	+	+	+	+	+	+	+	+	+	+	NA	+	+	+	+	+	+	NA	+	-	+	+	-	+
Sulaiman, 2022	+	+	+	+	+	+	+	+	+	-	+	+	+	NA	+	-	-	+	+	+	+	+	+	+	+	-	+
Lertsanguansinchaia, 2022	+	+	+	+	+	+	+	+	+	-	+	+	+	NA	NA	-	+	+	+	-	NA	+	+	+	+	-	+
Chen, 2023	+	+	-	+	+	-	+	-	-	-	NA	+	-	NA	NA	-	+	+	+	-	NA	-	-	+	+	+	-
Kwiecinski, 2023	+	+	+	+	+	+	+	+	+	-	NA	+	+	NA	NA	+	+	+	+	+	NA	+	+	+	+	+	+
Abdelkhalek, 2023	+	+	+	+	+	+	+	+	+	+	+	+	+	NA	NA	+	+	+	+	+	NA	+	+	+	+	-	+
Agasthi, 2023	+	+	+	+	+	+	+	-	+	+	+	+	+	NA	NA	-	+	+	+	+	NA	+	+	+	+	+	+
Leha, 2023	+	+	+	+	+	+	+	-	+	-	+	+	+	NA	+	+	+	+	+	+	NA	+	-	+	+	+	+
Alhwiti, 2023	+	+	+	+	+	+	+	-	+	+	+	+	+	NA	NA	-	+	+	+	+	NA	+	-	+	+	+	+
Pollari, 2023	+	-	+	+	+	+	+	+	+	-	+	+	+	NA	NA	-	-	-	-	+	NA	+	+	+	+	+	+
Savitz, 2023	+	+	+	+	+	+	+	+	+	-	+	+	+	NA	NA	-	-	+	+	+	NA	+	+	+	+	+	+
Barrett, 2023	+	+	+	+	+	+	+	+	+	-	+	+	+	NA	NA	+	+	+	+	+	NA	+	+	+	+	-	+
Theis, 2023	+	+	+	+	+	+	+	+	+	-	-	+	+	NA	NA	-	-	+	+	+	NA	+	+	+	+	+	+

**Stan, 2023**	+	+	+	+	+	+	+	+	+	-	-	+	+	NA	NA	-	-	+	+	+	+	+	+	+	+	+	+
**Ouahidi, 2024**	+	+	+	+	+	+	+	+	+	-	+	+	+	NA	-	+	+	+	+	+	NA	+	+	+	+	-	-
**Asif, 2024**	+	+	+	+	+	+	+	+	+	+	+	+	+	NA	NA	+	+	+	+	+	NA	+	+	+	+	-	+
**Barbieri, 2024**	+	+	+	+	+	+	+	+	+	-	+	+	+	NA	NA	+	+	+	+	+	NA	+	+	+	+	-	+
**Bruggemann, 2024**	+	+	+	+	+	+	+	+	+	-	+	+	+	NA	NA	+	+	+	+	+	NA	+	+	+	+	-	+
**Erck, 2024**	+	+	+	+	+	+	+	+	+	-	+	+	+	NA	NA	+	+	+	+	+	NA	+	-	+	+	-	+
**Erdogan, 2024**	+	+	+	+	+	+	+	+	+	-	+	+	+	NA	NA	+	+	+	+	+	NA	+	-	+	+	-	+
**Shi, 2024**	+	+	+	+	+	+	+	+	+	-	-	+	+	NA	NA	+	-	+	+	+	+	+	+	+	+	-	+
**Tremamunno, 2024**	+	+	+	+	+	+	+	+	+	-	-	+	+	NA	NA	+	-	+	+	+	NA	+	+	+	+	+	+
**Yordanov, 2024**	-	-	+	+	+	+	+	+	+	-	+	+	+	+	+	-	-	+	+	+	+	+	+	+	+	+	+
**Zahid, 2024**	+	-	+	+	+	+	+	+	+	-	+	+	+	NA	NA	+	+	+	+	+	+	+	+	+	+	+	+
**Zisiopoulou, 2024**	+	+	+	+	-	+	+	+	+	-	-	+	+	NA	NA	-	-	+	+	+	NA	+	+	+	+	+	+

Supplementary Table S3. Quality assessment of included studies with TRIPOD scoring [10,18-59].
