# Supplementary material for: Artificial Intelligence in Risk Stratification and Outcome Prediction for Transcatheter Aortic Valve Replacement: A Systematic Review and Meta-Analysis
Source: J Pers Med. 2025 Jul 11;15(7):302. doi: 10.3390/jpm15070302 (PMC12298983; doi:10.3390/jpm15070302)
Supplement: Supplementary file 1 [file jpm-15-00302-s001.zip › Supplementary Table S4.pdf]

Study	PROBAST Domains						
	Risk of bias Participants	Applicability Participants	Risk of bias Outcome	Applicability Outcome	Risk of bias Analysis	Overall risk of bias	Overall applicability
**Zusman, 2017**	Low concernn	Low concern	Low concern	Low concern	Low concern	Low concern	Low concern
**Vejpongsa, 2018**	Low concern	Low concern	Low concern	High concern	Low concern	Low concern	High concern
**Hoffmann, 2019**	Low concern	Low concern	Low concern	Low concern	Low concern	Low concern	Low concern
**Lopes, 2019**	High concern	Low concern	Low concern	Low concern	High concern	High concern	Low concern
**Hernandez-Suarez, 2019**	Low concern	Low concern	Low concern	Low concern	Low concern	Low concern	Low concern
**Tsushima, 2020**	Low Concern	Low Concern	Low Concern	Low Concern	Low Concern	Low Concern	Low Concern
**Abdul Ghaffr, 2020**	Low concern	Low concern	Low concern	Low concern	Low concern	Low concern	Low concern
**Gomes, 2020**	Low concern	Low concern	Low concern	Low concern	Low concern	Low concern	Low concern
**Truong, 2021**	Low Concern	Low Concern	Low Concern	Low Concern	High concern	High concern	Low Concern
**Agasthi, 2021**	Low concern	Low concern	Low concern	Low concern	High concern	High concern	Low concern
**Galli, 2021**	Low concern	Low concern	Low concern	Low concern	High concern	High concern	Low concern
**Penso, 2021**	Low concern	Low concern	Low concern	Low concern	High concern	High concern	Low concern
**Lopes, 2021**	Low concern	Low concern	Low concern	Low concern	Low concern	Low concern	Low concern
**Okuno, 2021**	High concern	Low concern	Low concern	Low concern	High concern	High concern	Low concern
**Mamprin, 2021 A**	High concern	Low concern	High concern	High concern	High concern	High concern	High concern
**Mamprin, 2021 B**	Low concern	Low concern	Low concern	Low concern	Low concern	Low concern	Low concern
**Sulaiman, 2022**	Low Concern	Low Concern	Low Concern	Low Concern	High Concern	High Concern	Low Concern
**Evertz, 2022**	Low concern	Low concern	Low concern	Low concern	High concern	High concern	Low concern
**Bansal, 2022**	Low concern	Low concern	Low concern	Low concern	High concern	High concern	Low concern
**Aquino, 2022**	Low concern	Low concern	Low concern	Low concern	Unclear	Unclear	Low concern
**Lertsanguansinchaia, 2022**	Low concern	Low concern	Low concern	Low concern	High concern	Low concern	High concern
**Abdelkhalek, 2023**	Low concern	Low concern	Low concern	Low concern	High concern	High concern	Low concern
**Pollari, 2023**	Low Concern	Low Concern	Low Concern	Low Concern	High Concern	High Concern	Low Concern
**Savitz, 2023**	Low Concern	Low Concern	Low Concern	Low Concern	High Concern	High Concern	Low Concern
**Stan, 2023**	Low Concern	Low Concern	Low Concern	Low Concern	Low Concern	Low Concern	Low Concern
**Theisa, 2023**	Low Concern	Low Concern	Low Concern	Low Concern	Low Concern	Low Concern	Low Concern
**Agasthi, 2023**	Low concern	Low concern	Low concern	Low concern	High concern	High concern	Low concern
**Alhwiti, 2023**	Low concern	Low concern	Low concern	Low concern	Low concern	Low concern	Low concern
**Barrett, 2023**	Low concern	Low concern	Low concern	Low concern	High concern	High concern	Low concern
**Chen, 2023**	Low concern	Low concern	Low concern	High concern	Low concern	Low concern	High concern
**Kwiecinski, 2023**	Low concern	Low concern	Low concern	Low concern	High concern	High concern	Low concern
**Leha, 2023**	Low concern	Low concern	Low concern	Low concern	High concern	High concern	Low concern
**Asif, 2024**	Low concern	Low concern	Low concern	Low concern	Low concern	Low concern	Low concern
**Shi, 2024**	Low Concern	Low Concern	Low Concern	Low Concern	Low Concern	Low Concern	Low Concern
**Tremamunno, 2024**							

	Low Concern	Low Concern	Low Concern	Low Concern	Low Concern	Low Concern	Low Concern
**Yordanov, 2024**	Low Concern	Low Concern	Low Concern	Low Concern	High Concern	High Concern	Low Concern
**Zahid, 2024**	Low Concern	Low Concern	Low Concern	Low Concern	High Concern	High Concern	Low Concern
**Barbieri, 2024**	Low concern	Low concern	Low concern	Low concern	High concern	High concern	Low concern
**Bruggemann, 2024**	Low concern	Low concern	Low concern	Low concern	High concern	High concern	Low concern
**Erck, 2024**	Low concern	Low concern	Low concern	Low concern	High concern	High concern	Low concern
**Erdogan, 2024**	Low concern	Low concern	Low concern	Low concern	High concern	High concern	Low concern
**Ouahidi, 2024**	Low concern	Low concern	Low concern	Low concern	Low concern	Low concern	Low concern
**Zisiopoulou, 2024**	Low Concern	Low Concern	Low Concern	Low Concern	Low Concern	Low Concern	Low Concern
**Number of H. concern**	3	0	1	3	24	23	4

Supplementary Table S4. Quality assessment of included studies with PROBAST scoring [10,18-59].
