# Supplementary figures and images for: Artificial Intelligence in Risk Stratification and Outcome Prediction for Transcatheter Aortic Valve Replacement: A Systematic Review and Meta-Analysis
Source: J Pers Med. 2025 Jul 11;15(7):302. doi: 10.3390/jpm15070302 (PMC12298983; doi:10.3390/jpm15070302)

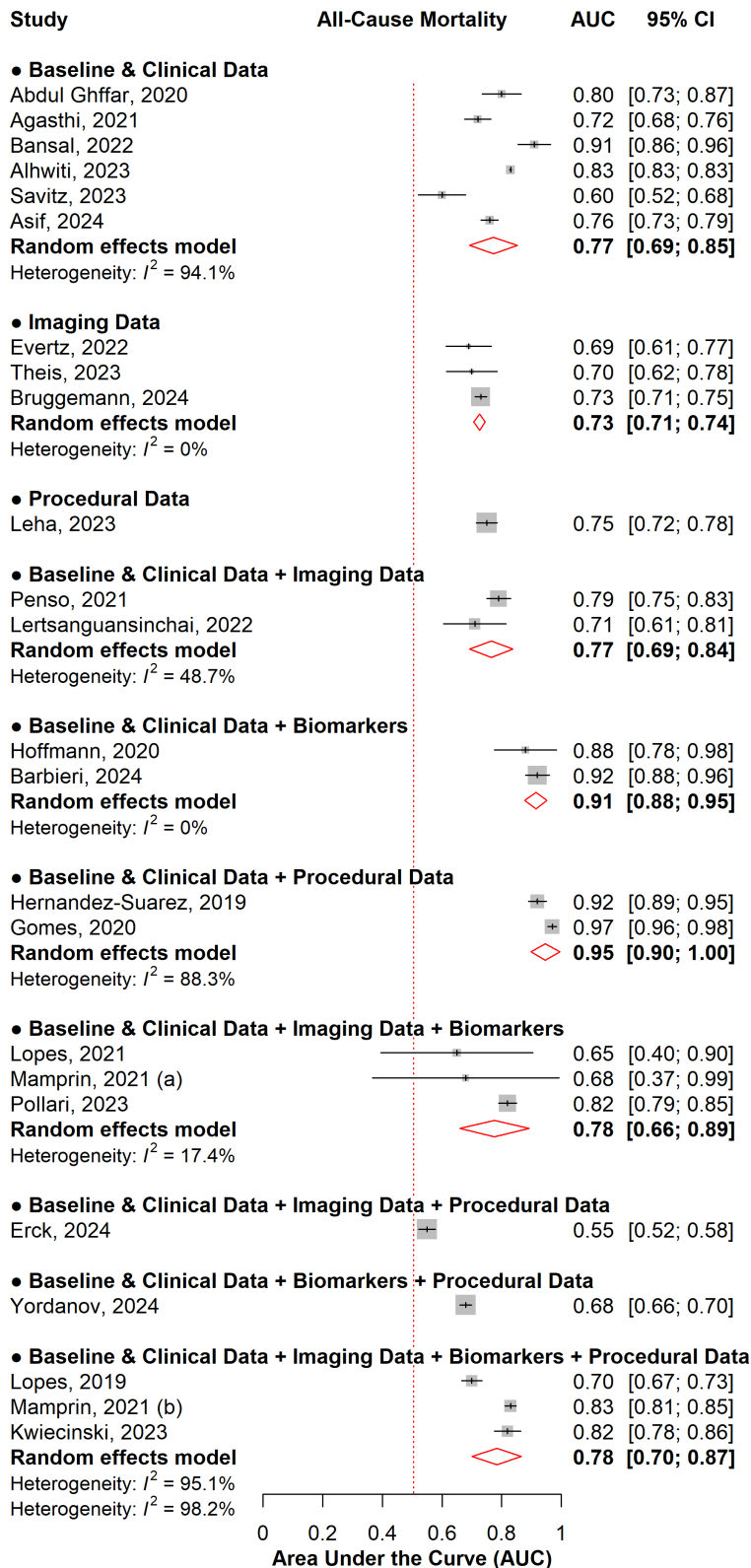

Supplement: Supplementary file 1 [file jpm-15-00302-s001.zip › Supplementary Figure S1.pdf]

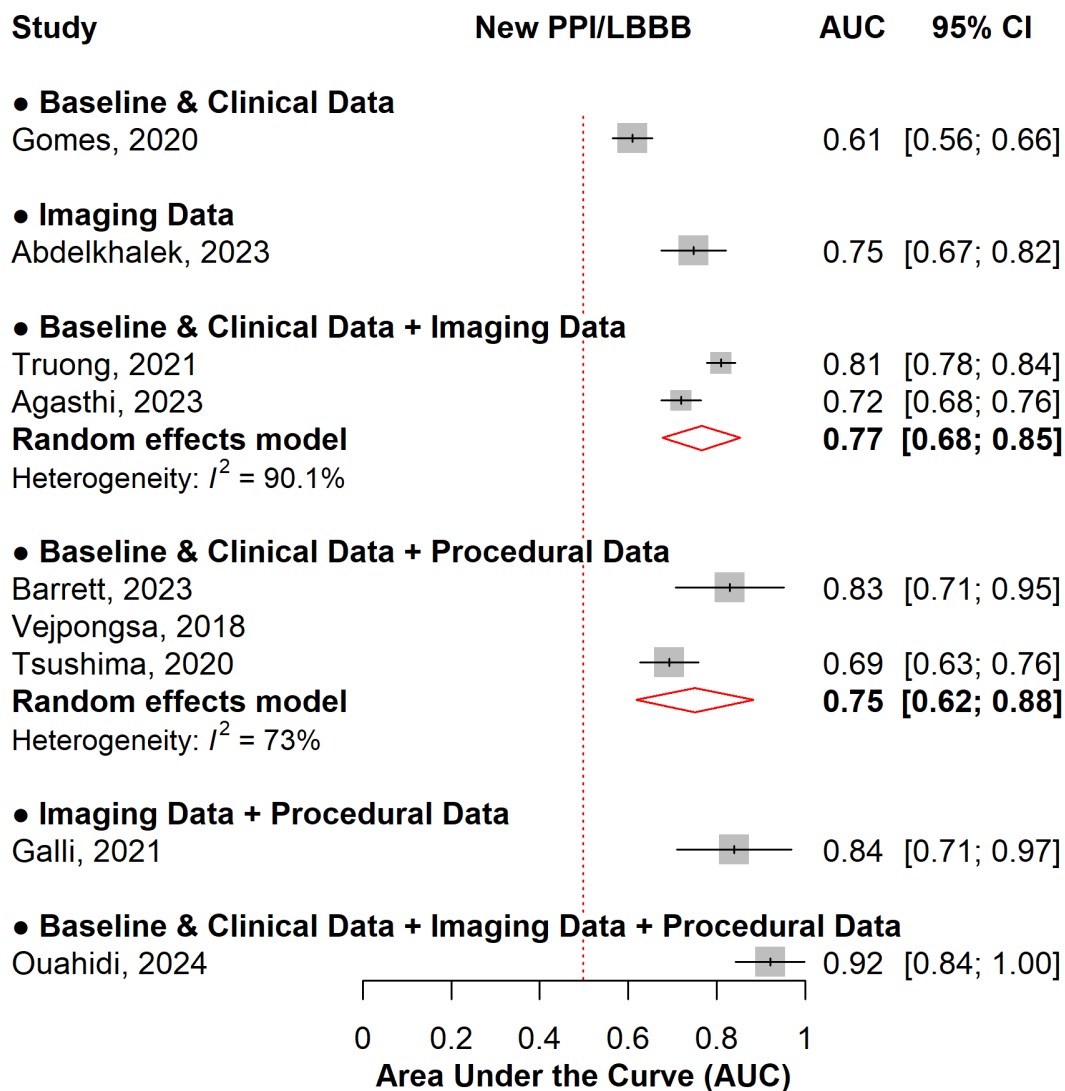

Supplement: Supplementary file 1 [file jpm-15-00302-s001.zip › Supplementary Figure S2.pdf]

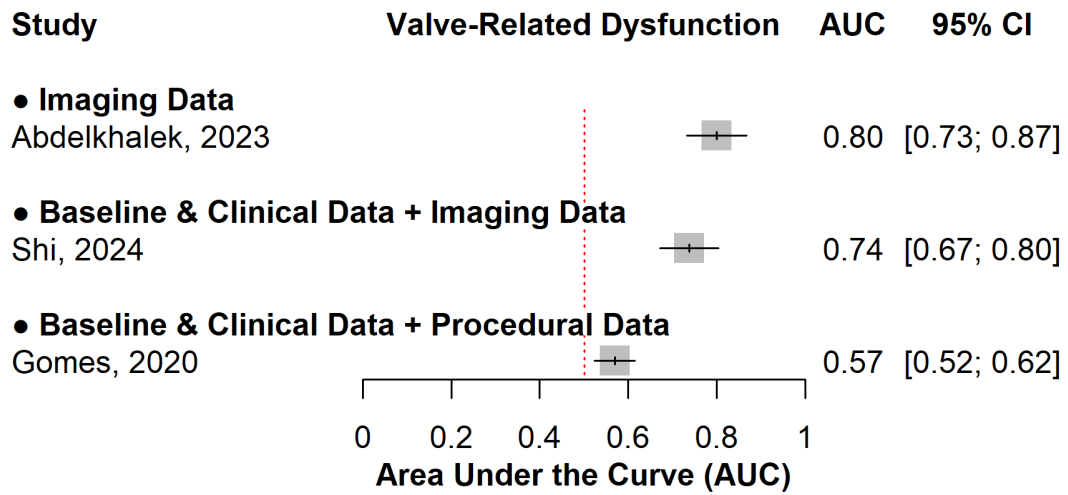

Supplement: Supplementary file 1 [file jpm-15-00302-s001.zip › Supplementary Figure S3.pdf]

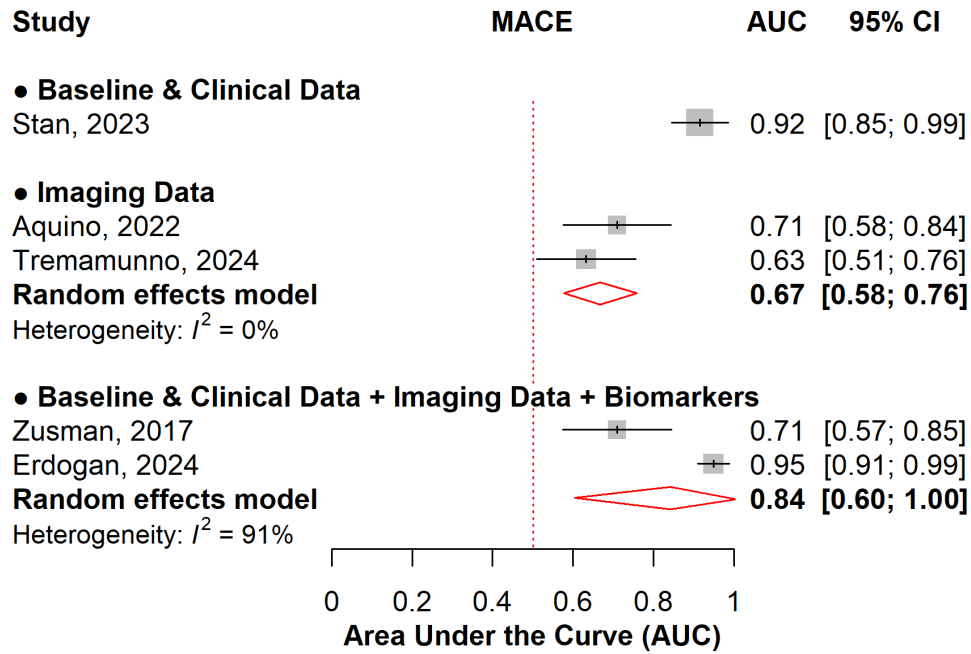

Supplement: Supplementary file 1 [file jpm-15-00302-s001.zip › Supplementary Figure S4.pdf]

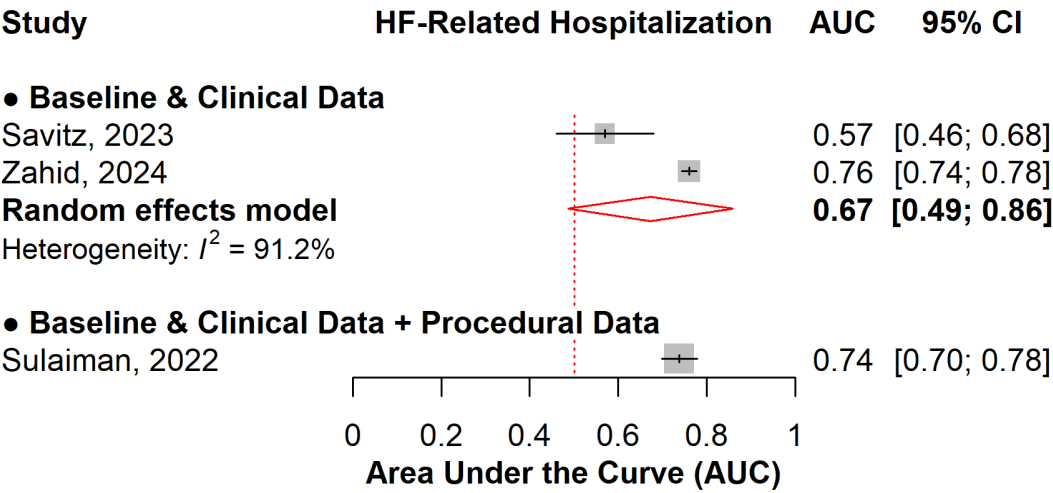

Supplement: Supplementary file 1 [file jpm-15-00302-s001.zip › Supplementary Figure S5.pdf]

# All-Cause Mortality

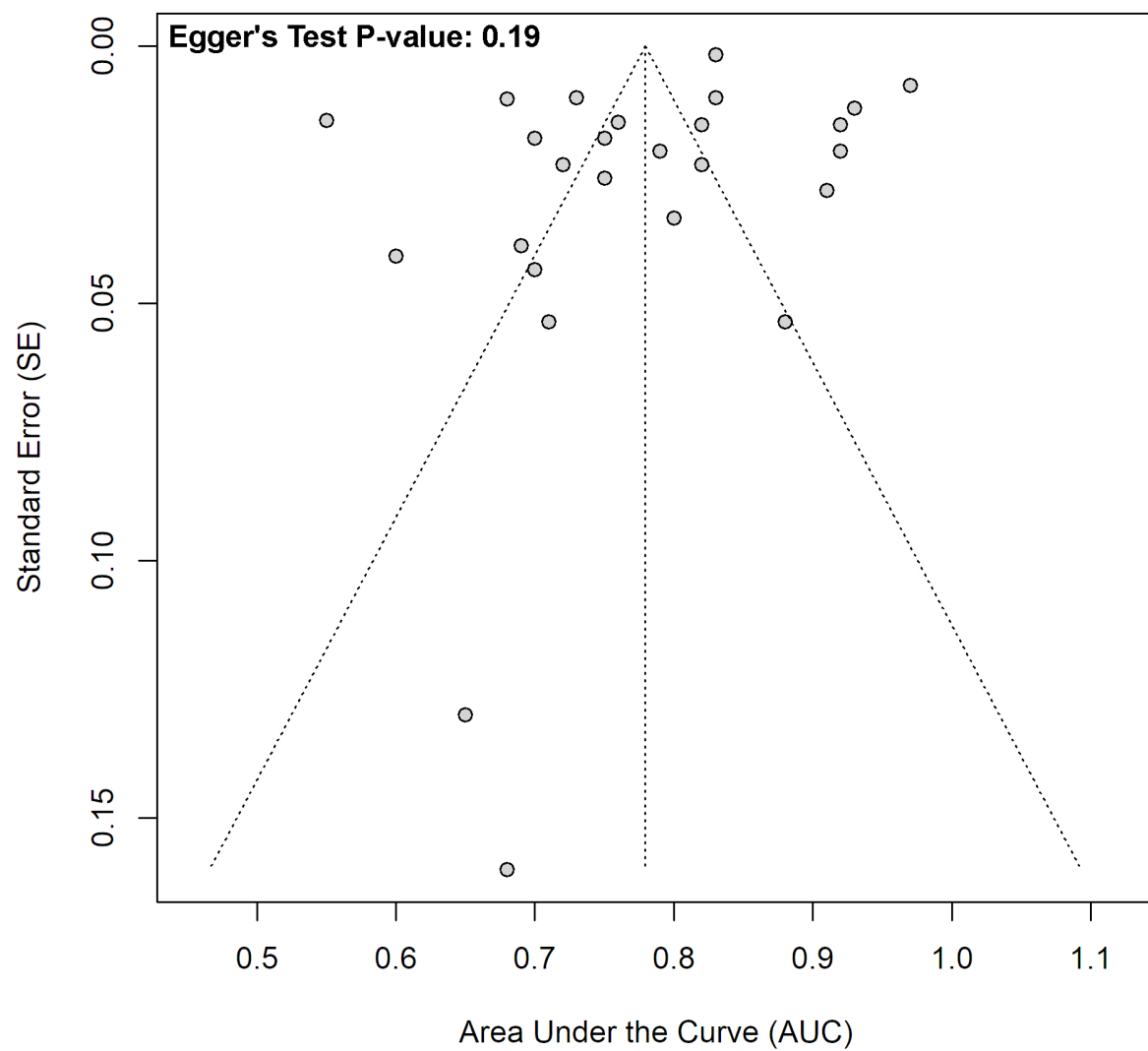

Supplement: Supplementary file 1 [file jpm-15-00302-s001.zip › Supplementary Figure S6.pdf]

# New PPI/LBBB

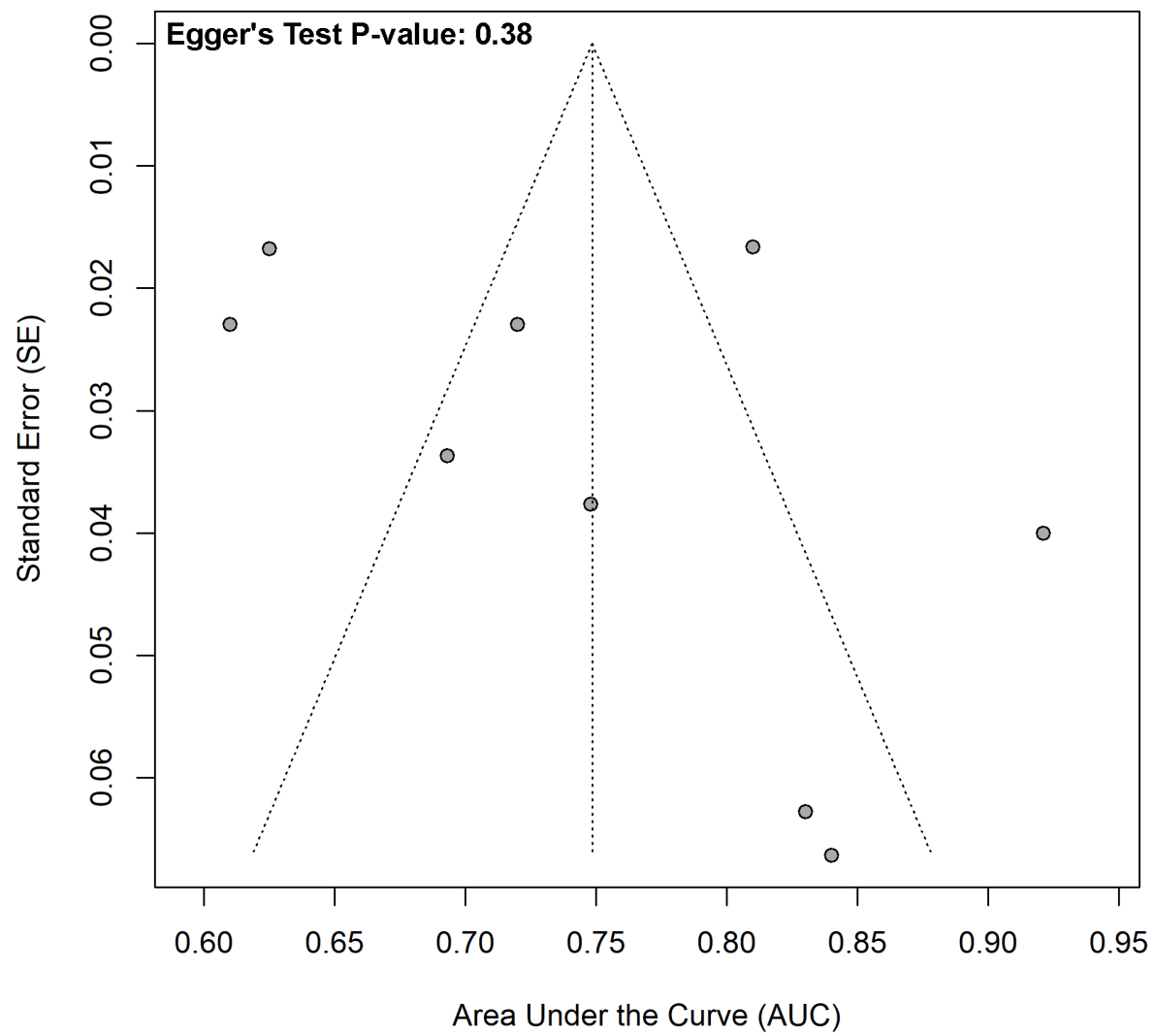

Supplement: Supplementary file 1 [file jpm-15-00302-s001.zip › Supplementary Figure S7.pdf]
